# Lean mass deposition occurs at a greater rate than fat deposition during pre-breeding stopover in highly depleted songbirds in the northern Gulf of Mexico

**DOI:** 10.1093/conphys/coaf029

**Published:** 2025-04-16

**Authors:** Mariamar Gutierrez Ramirez, Michael S Griego, Joely G DeSimone, Cory R Elowe, Alexander R Gerson

**Affiliations:** Department of Biology, University of Massachusetts Amherst, 611 N Pleasant St., 322 Morrill III, Amherst, MA 01003, USA; Organismic and Evolutionary Biology Program, University of Massachusetts Amherst, 611 N Pleasant St., 322 Morrill III, Amherst, MA 01003, USA; Department of Biology, University of Massachusetts Amherst, 611 N Pleasant St., 322 Morrill III, Amherst, MA 01003, USA; Organismic and Evolutionary Biology Program, University of Massachusetts Amherst, 611 N Pleasant St., 322 Morrill III, Amherst, MA 01003, USA; Department of Biology, University of Massachusetts Amherst, 611 N Pleasant St., 322 Morrill III, Amherst, MA 01003, USA; Department of Biology, University of Massachusetts Amherst, 611 N Pleasant St., 322 Morrill III, Amherst, MA 01003, USA; Organismic and Evolutionary Biology Program, University of Massachusetts Amherst, 611 N Pleasant St., 322 Morrill III, Amherst, MA 01003, USA; Department of Biology, University of Massachusetts Amherst, 611 N Pleasant St., 322 Morrill III, Amherst, MA 01003, USA; Organismic and Evolutionary Biology Program, University of Massachusetts Amherst, 611 N Pleasant St., 322 Morrill III, Amherst, MA 01003, USA

**Keywords:** Body condition, Gulf of Mexico, migratory songbird, quantitative magnetic resonance, stopover

## Abstract

The Gulf of Mexico represents the largest ecological barrier between breeding and non-breeding grounds for long-distance migratory songbirds in the Nearctic–Neotropical system. Despite the prominence of the Gulf of Mexico, there are still gaps on fundamental physiological aspects of stopover of migrants in this region, including the role and relative importance of fat and lean mass depletion and deposition. We examined the arrival body condition of Nearctic–Neotropical migrants at a coastal stopover site on St. George Island, FL, in the northern Gulf of Mexico during pre-breeding migration in the spring of 2016–2018. We precisely determined lean body and fat masses on individual birds after a trans-Gulf migratory flight via quantitative magnetic resonance (QMR) technology. We hypothesized that birds with non-breeding ranges in South America would arrive with lower fat and lean masses than birds with non-breeding ranges in the Caribbean or Central America. We also hypothesized that songbirds would increase lean mass at a greater rate than fat mass, as they rebuilt muscle and organ masses. We also compared QMR lean and fat measurements to visual measures of fat and muscle scores. A total of 44 Nearctic–Neotropical migratory bird species occur on St. George Island during spring stopover. Non-breeding range did not influence the arrival fat mass or arrival lean mass in 10 focal transient species, meaning those that have no breeding or non-breeding populations on the site. Our results from recaptured individuals indicated that body mass increase during stopover derives from both lean and fat mass accumulation. Our results provide a robust quantitative assessment of songbird arrival body condition on the northern Gulf of Mexico and contribute to the understanding of the physiology of migratory songbirds after a long-distance flight, which will help inform management decisions for stopover sites located around ecological barriers.

## Introduction

Long-distance migration allows migratory birds to exploit predictable seasonal changes in resource availability throughout the annual cycle. During their journeys, migratory songbirds must navigate many geographic and ecological barriers, and the decisions made *en route* can dramatically impact migration timing and strategies (Deppe *et al.,* 2015; Zenzal *et al.,* 2021). In preparation for non-stop over-water flights, songbirds must adequately deposit sufficient fat (Marsh, 1983; Ramenofsky, 1990) and increase pectoral muscle size (Marsh, 1984; Piersma, 1990; Hua *et al.,* 2013).

The Gulf of Mexico, with 800–1000 km of open water, is the most conspicuous geographical feature in the Nearctic–Neotropical migratory system (Cohen *et al.,* 2017), and successfully crossing this barrier may be a key limiting factor to surviving migration. Over 2 billion birds pass over the Gulf of Mexico every spring *en route* to breeding areas across North America from the non-breeding grounds in Mexico, the Caribbean and Central and South America (Horton *et al.,* 2019). With the assistance of favourable winds, which are more common in spring (Clipp *et al.,* 2020), a trans-Gulf of Mexico flight can take an average of 15 hours of non-stop flight (Gauthreaux Jr, 1999), while a circum-Gulf flight can increase migration duration up to 21% (Abdulle and Fraser, 2018). Though quicker than a circum-Gulf route, a trans-Gulf of Mexico flight is not without risk. Many birds do not make it across the Gulf successfully, as there are reports of songbirds washing up on the coastlines (Moore *et al.,* 1990) and found inside the stomach of tiger sharks (*Galeocerdo cuvier*) (Drymon *et al.,* 2019). Among those that cross successfully, many arrive with depleted fat reserves and concave pectoral muscles (Moore and Kerlinger, 1987; Kuenzi *et al.,* 1991; Yong and Moore, 1997), in a dehydrated state (Leberg *et al.,* 1996), and are subjected to increased predation risk (Moore *et al.,* 1990). Winds over the Caribbean Sea strongly influence stopover density in the eastern Gulf of Mexico (Clipp *et al.,* 2021), which has led others to suggest some songbirds may undertake a direct flight from South America via a trans-Caribbean route (Lafleur *et al.,* 2016; Cano *et al.,* 2020). However, in the eastern portion of the northern Gulf of Mexico, songbirds regularly arrive in very poor body condition and have low refuelling rates, long periods of inactivity and short stopover duration (Kuenzi *et al.,* 1991; Aborn and Moore, 2004; Gutierrez Ramirez *et al.,* 2021). This may suggest that songbirds alighting in the eastern Gulf of Mexico may incur a higher cost than those moving through the western and central parts of the Gulf.

Poor body condition after crossing an ecological barrier can significantly decrease the pace of migration because the rate of fuel deposition at stopover is physiologically limited (Carpenter *et al.,* 1993; Karasov and Pinshow, 2000; Gannes, 2002). The limited rate of fuel deposition is a result of muscle and organ mass loss incurred during flight (Kuenzi *et al.,* 1991; Karasov and Pinshow, 1998; Lindström *et al.,* 2000; Maggini and Bairlein, 2011). In some species, individuals with lower lean mass have prolonged stopover duration after crossing the Gulf of Mexico (Gutierrez Ramirez *et al.,* 2022), likely attributable to the need for increased recovery time.

To understand lean body mass dynamics during stopover, we captured songbirds during spring (hereafter, ‘pre-breeding’; Albert and Siegel, 2024) migration 2016–2018 and measured body composition using quantitative magnetic resonance (QMR) technology to accurately and non-destructively measure fat and lean mass (Guglielmo *et al.,* 2011) of migratory songbirds that had just completed a trans-Gulf flight and arrived on a barrier island (St. George Island, FL, USA, 29.672679, −84.841423). We established three major goals for this study. First, we use QMR and traditional visual measures of fat and muscle scores to describe the body condition of migratory passerines alighting on the island. We compare QMR measurements to fat and muscle scoring, aiming to calibrate and facilitate comparisons of body condition with other studies.

Second, we test the hypothesis that the body composition of migratory songbirds arriving after crossing the Gulf of Mexico during pre-breeding migration would reflect general distance from the non-breeding site to the stopover. We expected birds that spend the non-breeding season in South America would arrive with lower fat and lean masses than birds that spend the non-breeding season in the Caribbean or Central America, since songbirds that depart from the Caribbean and Central America traverse a shorter distance and do not have to cross the Caribbean Sea in addition to the Gulf of Mexico, which may be completed as a direct non-stop migratory flight from South America (Lafleur *et al.,* 2016; Cano *et al.,* 2020).

Third, we test the hypothesis that lean mass increases at a greater rate than fat mass because songbirds must rebuild muscle and organ masses before accumulating fat reserves required to resume migration (Carpenter *et al.,* 1993; Karasov and Pinshow, 1998; Wojciechowski *et al.,* 2014). We expected that individuals stopping over on the island would show increases in non-fat components with subsequent recaptures, since most birds arrive with depleted fat reserves. To address this hypothesis, we used QMR to measure fat and lean deposition rates of spring migrants based on recaptured individuals.

## Materials and Methods

### Study site

**Figure 1:**
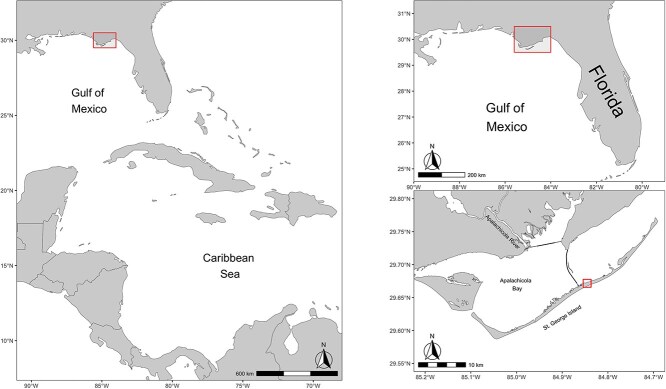
Map of St. George Island, Apalachicola Bay, FL, in relation to the Gulf of Mexico and Caribbean Sea. Top right map shows the location of the Apalachicola Bay, St. George Island in northern Florida, USA. Bottom right map shows the banding location on St. George Island in the square.

This study was conducted on the northeastern Gulf of Mexico, on St. George Island, FL, a narrow 33-km-long barrier island located on the southern edge of Apalachicola Bay (Fig. 1). St. George Island is approximately 7 km from the mainland and is the first landfall opportunity for migrants crossing the Gulf of Mexico during pre-breeding migration (Lester *et al.,* 2016; Gutierrez Ramirez *et al.,* 2021). The vegetation on St. George Island consists of a narrow band of beaches and low-lying sand dunes that grade into mixed woodland grass, palmetto and bayside marshes (Edmiston, 2008). Field data were collected on public land managed by the Apalachicola National Estuarine Research Reserve and adjacent private property consisting of patches of coastal pine forest on the sound side of the island.

### Bird capture, measurements and body composition

We operated a constant-effort mist-netting station from 6 April to 6 May 2016, 1 April to 12 May 2017 and 2 April to 6 May 2018 consisting of 10 to 16 mist-nets (6- and 12-m length × 2.6-m height and 38-mm mesh). Mist-nets were opened daily at sunrise, unless rain or steady winds (>10 mph) prevented safe operation, and closed at ~12:00 eastern daylight time (EDT) to avoid high temperatures and decreased shade. In 2017 and 2018, mist-nets were also opened in the evening (~16:00 EDT) and closed prior to sunset. Total effort was 1594 net/hours in 2016, 2618 net/hours in 2017 and 1954 net/hours in 2018. Nets were checked every 10 minutes. Upon capture, birds were safely and quickly extracted from mist-nets and transferred into a cloth bag.

All captured birds were identified to species and banded with an individually numbered US Geological Survey aluminium band. Birds were aged and sexed when possible as second year or after second year (Pyle, 1997). We used a wing ruler to measure unflattened wing chord and tail length, and digital callipers (to the 0.01 mm) to measure tarsus, nares to tip, exposed culmen, bill width and sternum length. We visually scored fat on a 9-point scale (Kaiser, 1993) and pectoral muscle on a 4-point scale (Bairlein, 1995). QMR is a non-lethal, non-invasive technique for measuring lean, lipid and water mass (Guglielmo *et al.,* 2011) that can be used under field conditions. We scanned birds at least twice for fat mass, lean mass and total water using a QMR body composition analyser (EchoMRI-B, Echo Medical Systems, Houston, USA) in the field, with a total scanning time of up to 180 seconds. All birds were scanned in the QMR upon initial capture. All individuals were scanned upon recapture, except for a few species that were radio-tagged for a separate, concurrent study (Gutierrez Ramirez *et al.,* 2022). Lean mass was corrected for body size (wing chord; Gosler *et al.,* 1998) using a scaled-mass model (Peig and Green, 2009) in species where a simple linear regression of lean mass and body size was significant (*P* < 0.05). Lean body mass was highly correlated with body size in Swainson’s Thrush (*Catharus ustulatus*; SWTH) (wing: *F*_1,26_ = 9.04, *P* = 0.005) and in Hooded Warbler (*Setophaga citrina*; HOWA) after accounting for sex (wing × sex(F): *t* = −3.425, *P* = 0.002). Therefore, for these species we calculated scaled lean mass (g) as a measure of lean mass corrected for size. For all other species, we used uncorrected lean mass measurements. Fat mass was independent of size in each species (all *P >* 0.1), so we did not make corrections to fat mass measurements.

### Blood sample for sex determination

Because stopover phenology and other aspects of stopover behaviour are known to vary by sex (Seewagen *et al.,* 2013; Hays *et al.,* 2018; Morbey *et al.,* 2018), a blood sample was collected (<1% of estimated blood volume, Fair *et al.,* 2010) from each individual by brachial venipuncture to determine the sex of monomorphic species. Blood samples were stored on ice in the field for up to 6 h and then centrifuged at 1000 rpm for 10 min to separate plasma from red blood cells. Red blood cells were stored at −20°C until DNA was extracted (InstaGene Matrix, BioRad) in preparation for polymerase chain reaction (PCR) using P2/P8 primers (Griffiths *et al.,* 1998). We used gel electrophoresis (2% agarose gel, 120 V for 60 minutes) to visualize PCR products and assigned individuals as male if one band developed or female if two bands developed.

Bird capture, blood sampling and body composition analysis were conducted under appropriate permits from the Florida Fish and Wildlife Conservation Commission (Permit LSSC-16-00033) and the US Geological Survey, Bird Banding Laboratory (permit 23 979), and approved by the Institutional Animal Care and Use Committee at the University of Massachusetts Amherst (2015–0019).

### Focal species and classification

A species was selected as a focal species if it was a Nearctic–Neotropical migratory species with no confirmed breeding or non-breeding populations on the island and had high passage density through the stopover site (*n* > 20 individuals over the three-year period). The 10 focal species were Blue Grosbeak (*Passerina caerulea*; BLGR), Indigo Bunting (*Passerina cyanea*; INBU), Red-eyed Vireo (*Vireo olivaceus*; REVI), Summer Tanager (*Piranga rubra*; SUTA), Northern Waterthrush (*Parkesia noveboracensis*; NOWA), Ovenbird (*Seiurus aurocapilla*; OVEN), Hooded Warbler, Swainson’s Thrush, Veery (*Catharus fuscescens*; VEER) and Wood Thrush (*Hylocichla mustelina*; WOTH) (see Table S1 for sample size per year). Focal species were classified based on the non-breeding range as primarily Caribbean-Central America, Central America-northern South America, or South America regions (based on species ranges).

### Data analysis

We describe the body condition of migratory birds on stopover using the averaged QMR measurements of fat and lean mass values (all coefficient of variation <15%). We calculated the median for visual scores of fat and muscle, as this is the most appropriate measure of central tendency for non-normal variables; however, we also report the mean and standard error since this is more commonly reported in other studies. We used linear models separately for each species to examine the relationship between visual scores of body condition (fat and muscle scores, explanatory variables) and QMR measurements (fat and lean masses, response variables).

To address our hypothesis of differential arrival body condition linked to general non-breeding range, we modelled arrival body condition (fat and lean masses) as a function of non-breeding range (South America, Caribbean/Central America, or Central America/northern South America), ordinal day, year; species was included as a random factor.

To address the hypothesis that birds increase lean mass during stopover as they rebuild muscle and organ masses, we calculated the change in fat mass and lean mass in focal individuals recaptured >1 day after initial capture. We conservatively calculated length of stopover as the difference between first and last capture of an individual, assuming these capture events corresponded with arrival and departure, respectively, and used this to calculate change in grams per day. The change in fat mass per day and change in lean mass per day were compared using *t*-test. All analyses were conducted with the program R version 4.0.1 (R Core Team, 2019). We used R packages ‘lme4’ (Bates *et al.,* 2015) for building mixed models.

**Table 1:** The average (±SD) and range of body composition, wing chord, fat score, muscle score and the sex ratio of transient migratory birds during spring stopover on St. George Island, FL, from 2016 to 2018

	Fat (g)	Lean (g)	Body mass (g)	Wing (mm)	Fat score	Muscle score	Sex, M/F/U
Blue Grosbeak							
2016 (1)	0.03 ± NA 0.03–0.03	19.38 ± NA 19.38–19.38	26.27 ± NA 26.27–26.27	91.00 ± NA 91.00–91.00	2.00 ± NA [2.00]	2.00 ± NA [2.00]	1/0/0
2017 (30)	1.32 ± 1.25 0.07–4.23	19.20 ± 2.53 10.31–23.47	24.99 ± 3.10 20.42–32.65	84.93 ± 3.35 79.00–92.00	1.87 ± 0.88 [2.00]	2.16 ± 0.45 [2.00]	14/16/0
2018 (9)	5.70 ± 4.01 0.00–10.33	20.57 ± 1.40 18.02–21.95	29.92 ± 5.11 21.61–34.83	85.89 ± 3.14 80.00–88.00	3.67 ± 1.58 [4.00]	3.11 ± 0.60 [3.00]	7/2/0
Hooded Warbler							
2016 (3)	0.73 ± 0.11 0.62–0.84	4.99 ± 0.88 4.11–5.86	9.23 ± 0.82 8.67–10.17	63.33 ± 2.08 61.00–65.00	1.00 ± 0.00 [1.00]	2.67 ± 0.58 [3.00]	1/2/0
2017 (3)	0.79 ± 1.06 0.14–2.02	7.23 ± 0.56 6.58–7.62	9.61 ± 1.22 8.50–10.92	62.33 ± 2.52 60.00–65.00	1.25 ± 0.50 [1.00]	2.75 ± 0.96 [2.50]	1/2/0
2018 (22)	0.45 ± 0.45 0.00–1.50	7.43 ± 0.53 6.38–8.52	9.50 ± 0.85 7.96–11.51	64.86 ± 3.27 58.00–70.00	1.38 ± 0.68 [1.00]	2.62 ± 0.68 [3.00]	18/4/0
Indigo Bunting							
2016 (8)	0.97 ± 0.56 0.49–1.91	8.72 ± 1.06 6.71–9.62	13.25 ± 1.09 11.44–14.64	65.50 ± 2.62 60.00–68.00	1.88 ± 0.83 [2.00]	3.12 ± 0.64 [3.00]	6/2/0
2017 (26)	1.01 ± 1.00 0.02–3.42	9.09 ± 1.26 7.00–11.99	12.84 ± 1.46 10.96–15.42	65.27 ± 2.79 60.00–70.00	2.00 ± 1.33 [1.00]	2.27 ± 0.67 [2.00]	16/10/0
2018 (19)	0.48 ± 0.41 0.04–1.66	9.87 ± 1.06 7.12–11.75	12.61 ± 1.24 10.84–15.34	65.79 ± 2.35 63.00–70.00	1.88 ± 0.85 [2.00]	2.83 ± 0.64 [3.00]	12/7/0
Northern Waterthrush							
2016 (31)	1.38 ± 1.35 0.13–5.38	10.07 ± 1.98 6.45–13.08	15.00 ± 1.97 12.63–19.79	74.03 ± 2.68 70.00–81.00	2.38 ± 1.24 [2.00]	2.66 ± 0.70 [3.00]	21/10/0
2017 (16)	1.54 ± 1.40 0.21–4.99	11.54 ± 1.13 9.73–13.24	15.28 ± 1.81 12.42–18.35	74.81 ± 3.33 70.00–84.00	2.12 ± 1.27 [2.00]	2.06 ± 0.66 [2.00]	14/1/1
2018 (5)	2.38 ± 1.92 0.72–5.33	10.13 ± 0.77 9.12–11.14	14.93 ± 1.72 13.25–17.39	71.00 ± 2.35 69.00–74.00	2.60 ± 1.34 [2.00]	2.80 ± 0.84 [3.00]	4/0/1
Ovenbird							
2016 (20)	0.95 ± 0.83 0.00–3.71	12.22 ± 1.43 8.89–15.47	16.58 ± 1.32 14.83–19.86	74.10 ± 3.55 68.00–82.00	2.05 ± 0.86 [2.00]	2.57 ± 0.68 [3.00]	12/5/3
2017 (16)	1.09 ± 1.54 0.03–5.38	12.18 ± 2.28 6.29–14.51	16.70 ± 2.30 13.48–21.21	76.38 ± 4.27 68.00–86.00	1.94 ± 1.39 [1.00]	2.18 ± 0.53 [2.00]	10/2/4
2018 (13)	0.86 ± 1.00 0.00–3.22	12.37 ± 1.53 9.09–14.58	16.31 ± 1.07 14.55–18.16	73.92 ± 2.10 71.00–77.00	2.06 ± 1.26 [2.00]	2.28 ± 0.57 [2.00]	8/2/3
Red-eyed Vireo							
2016 (9)	0.70 ± 0.38 0.23–1.24	9.88 ± 1.48 7.43–11.56	14.40 ± 0.79 13.23–15.85	80.22 ± 2.17 76.00–83.00	1.67 ± 0.71 [2.00]	2.11 ± 0.33 [2.00]	2/2/5
2017 (11)	1.53 ± 1.84 0.10–5.87	11.33 ± 1.77 8.30–13.83	15.80 ± 2.63 13.35–21.64	78.45 ± 2.81 73.00–82.00	2.36 ± 1.03 [2.00]	2.64 ± 0.81 [2.00]	2/1/8
2018 (12)	1.53 ± 1.47 0.00–4.80	11.09 ± 1.25 9.13–12.80	15.02 ± 1.85 11.87–17.78	77.92 ± 3.37 74.00–85.00	2.36 ± 1.39 [2.00]	2.29 ± 0.91 [2.00]	1/0/11
Summer Tanager							
2016 (3)	0.89 ± 0.22 0.73–1.14	20.57 ± 0.50 20.00–20.92	26.49 ± 0.79 25.68–27.26	94.00 ± 2.00 92.00–96.00	1.33 ± 0.58 [1.00]	1.67 ± 0.58 [2.00]	2/1/0
2017 (8)	1.22 ± 0.57 0.40–2.42	20.58 ± 1.53 18.62–23.19	26.75 ± 1.73 23.78–28.79	91.75 ± 2.60 87.00–96.00	1.38 ± 0.74 [1.00]	2.12 ± 0.64 [2.00]	5/3/0
2018 (15)	1.62 ± 1.54 0.01–4.96	20.82 ± 1.55 18.45–23.24	26.58 ± 2.74 23.48–32.59	93.00 ± 2.56 89.00–99.00	1.67 ± 0.90 [1.00]	1.93 ± 0.70 [2.00]	13/3/0
Swainson’s Thrush							
2016 (4)	1.10 ± 1.59 0.00–3.42	20.05 ± 1.33 18.23–21.23	25.66 ± 2.45 22.75–28.36	100.50 ± 2.08 98.00–103.00	2.00 ± 0.82 [2.00]	2.00 ± 0.00 [2.00]	3/1/0
2017 (15)	1.22 ± 0.96 0.00–3.37	20.23 ± 1.45 17.30–22.67	25.55 ± 1.94 21.63–28.64	97.27 ± 3.01 92.00–102.00	1.87 ± 0.99 [2.00]	2.07 ± 0.59 [2.00]	9/5/1
2018 (9)	1.32 ± 0.77 0.00–2.54	20.54 ± 1.53 18.16–22.46	25.94 ± 1.54 23.61–27.92	98.44 ± 5.70 90.00–106.00	1.89 ± 0.60 [2.00]	2.22 ± 0.44 [2.00]	5/4/0
Veery							
2016 (3)	2.96 ± 0.66 2.54–3.72	20.59 ± 0.24 20.36–20.84	27.37 ± 0.55 26.84–27.93	96.00 ± 3.61 92.00–99.00	3.00 ± 0.00 [3.00]	2.33 ± 0.58 [2.00]	2/1/0
2017 (4)	1.94 ± 0.68 1.14–2.79	20.47 ± 1.67 18.30–22.16	26.50 ± 2.55 23.38–29.34	100.75 ± 6.99 94.00–110.00	2.50 ± 0.58 [2.50]	1.75 ± 0.50 [2.00]	2/2/0
2018 (15)	1.39 ± 1.35 0.03–4.71	19.26 ± 1.24 16.98–21.39	24.70 ± 2.21 20.97–29.66	96.20 ± 3.71 91.00–102.00	1.71 ± 0.77 [2.00]	1.88 ± 0.60 [2.00]	5/2/9
Wood Thrush							
2016 (9)	0.94 ± 0.71 0.16–2.10	29.17 ± 2.09 26.75–32.59	37.10 ± 2.29 33.81–40.56	105.00 ± 5.68 94.00–113.00	1.11 ± 0.33 [1.00]	1.78 ± 0.44 [2.00]	1/0/8
2017 (7)	1.81 ± 2.95 0.25–8.21	29.68 ± 2.78 23.75–32.49	38.74 ± 1.78 37.10–41.71	107.29 ± 5.65 101.00–118.00	1.57 ± 0.53 [2.00]	1.57 ± 0.53 [2.00]	2/2/3
2018 (21)	3.58 ± 3.66 0.18–13.81	31.93 ± 2.26 28.80–37.92	42.37 ± 4.33 36.53–52.39	105.33 ± 3.94 98.00–112.00	2.14 ± 1.28 [2.00]	2.27 ± 0.77 [2.00]	4/3/14

The median for fat and muscle scores in brackets. Fat mass and lean mass (g) were obtained by QMR scanning, body mass (g) was obtained by digital scale and wing chord (mm) was measured with a ruler. Sex was determined by plumage in dimorphic species and by molecular methods in monomorphic species. Some individuals remained unsexed. Sample sizes in parentheses.

## Results

We captured 68 species over the three pre-breeding migratory (spring) seasons on St. George Island. Nearctic–Neotropical migrants made up 65% of the species captured (44 species), and 51% were transient migrants (35 species), with no known breeding or non-breeding population on the island. A complete list of species captured on St. George Island is presented in Table S1. The average and range of body mass (g), fat mass (g) and lean mass (g) and the median of fat and muscle scores of focal species are listed in Table 1 and of all species captured in Table S2.

**Figure 2:**
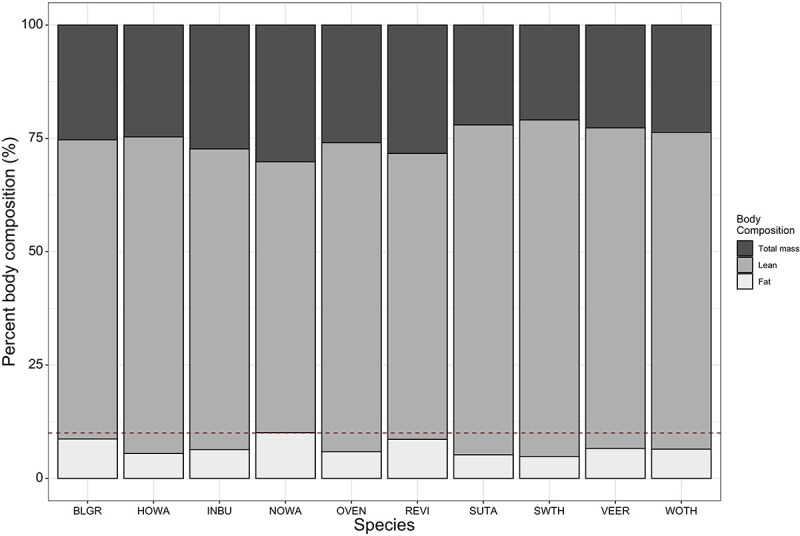
Body composition of 10 focal species during pre-breeding migration on the northern Gulf of Mexico. All birds arrived with body fat averaging <10% of body mass (dotted red line). Total mass represents whole animal mass as measured with digital scale, and includes all body components (bones, lean and fat mass, feathers); lean mass was measured via QMR and represents lean wet mass (muscle and organ tissues); and fat mass was measured via QMR and represents fat content.

**Figure 3:**
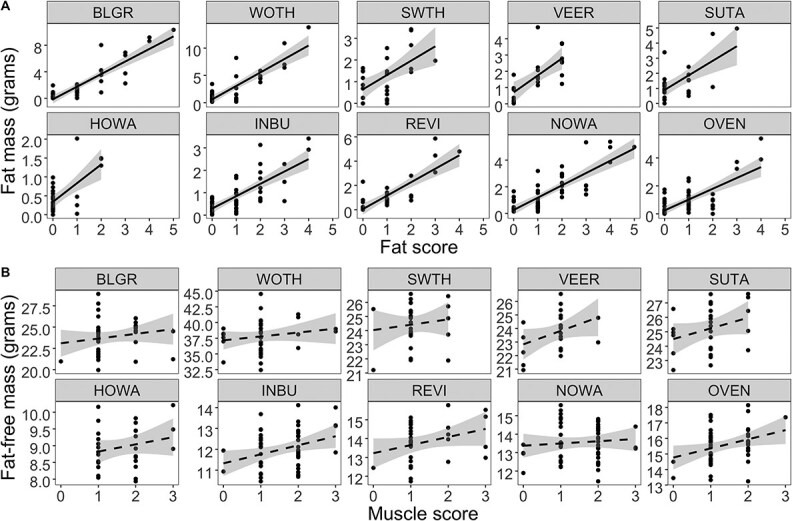
Correlation between QMR body composition analysis data for (**A**) fat mass and fat scores and (**B**) fat-free and pectoral muscle score of ten focal species of transient Nearctic–Neotropical migrants during spring stopover in St. George Island, FL.

Five focal species had a non-breeding range primarily in Central America—Caribbean: Blue Grosbeak, Hooded Warbler, Indigo Bunting, Ovenbird and Wood Thrush. Two focal species had a wide non-breeding range extending from Central America into northern South America: Northern Waterthrush and Summer Tanager. Three focal species had a non-breeding range primarily in South America: Red-eyed Vireo, Swainson’s Thrush and Veery. Average body fat on arrival for every focal species was <10% body mass (Fig. 2). Visual fat scores correlated positively with QMR fat mass and fat-free mass in all species (Fig. 3A), but there was no significant correlation between visual muscle scores and QMR fat-free mass measurements (Fig. 3B).

Non-breeding range did not influence the arrival fat mass or arrival lean mass (fat: *F*_2,9_ = 0.0156, *P* = 0.98; lean: *F*_2,9_ = 0.0297, *P* = 0.97). This was regardless of size differences between species, as species was included in the model as a random effect.

**Figure 4:**
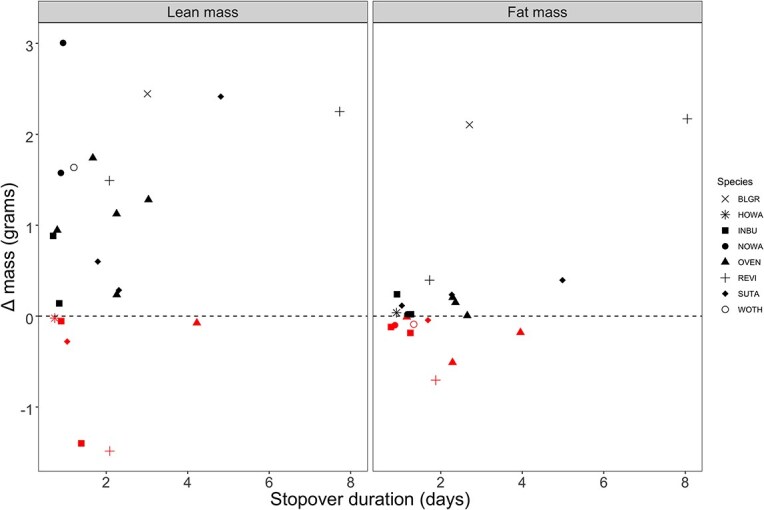
Rate of fat mass and lean mass change relative to minimum stopover duration of individual Neotropical migrants recaptured >1 day after first capture in St. George Island, FL, during pre-breeding migration 2016–2018. Fat and lean mass measured by QMR in the field. The dashed horizontal line indicates no change in mass between first capture and subsequent recapture, so that below the line are birds with negative rate and above the line are birds with positive rate of mass change.

We estimated daily rates of fat and lean mass changes and stopover duration for 25 individual birds that were recaptured >1 day after initial capture (Table 2). Time between captures ranged from 1 to 8 days. Daily rate of mass change was significantly greater for lean mass than fat mass (*t*_27_ = −2.6432, *P* = 0.01335; Fig. 4).

## Discussion

Our field study presents a comprehensive description of arrival body condition using QMR on Nearctic–Neotropical migratory songbirds after a non-stop migratory flight over open water. Body composition has been measured using QMR technology in actively migrating wild songbirds in previous studies (Seewagen and Guglielmo, 2010, 2011; Kennedy *et al.,* 2017; Kelsey *et al.,* 2021) but none along the Gulf of Mexico after a non-stop long-distance migratory flight (but see Gutierrez Ramirez *et al.,* 2022). We found that birds arrived with lower lean mass than previously estimated or recorded for many of these species. For example, the average lean mass for Northern Waterthrush on St. George Island (10.53 ± 1.8 g) was lower than the lean body mass estimated from individuals that had completed over-water flights during post-breeding migration in Colombia (14.1 ± 0.49 g) (Cano *et al.,* 2020), or directly measured from television tower causalities in Florida (13.95 ± 1.13 g) (Rogers and Odum, 1966). Comparing our body mass measurements to “unusually light post-migrants” from Panama during post-breeding migration, we found that Veery, Swainson’s Thrush, Summer Tanager and Red-eyed Vireo had less fat mass though similar body mass (Rogers and Odum, 1966). We recognize the difficulty of comparing QMR measurements of fat and lean masses with estimates of fat-free lean mass or lethal measures of body composition, mainly because QMR does not include water-free components (feather, bones, bill) in its measure of lean mass. However, our findings suggest that many migratory songbirds alighting in the eastern Gulf of Mexico are pushing their physiological limits, and these may be lower than previously reported. Muscle loss and under 5–10% body fat can result in reduced flight performance for migratory passerines (Schwilch *et al.,* 2002). This highlights the significance of non-fat components (lean mass) in addressing the needs of migratory birds during stopover and in assessing the management of stopover sites. In fact, the true value of stopover habitats to migrants should be assessed by the provision of water, sleep and protein, and not just increases in fat reserves (Linscott and Senner, 2021).

Contrary to previous studies in eastern Gulf of Mexico barrier islands during pre-breeding migration (Kuenzi *et al.,* 1991; DeSimone *et al.,* 2020; Gutierrez Ramirez *et al.,* 2021), we found that recaptured birds tended to gain mass. The increase in body mass prior to migration has been measured via QMR as primarily fat mass gain and not a change in dry lean mass (Seewagen and Guglielmo, 2011; Kelsey and Bairlein, 2019). However, we show evidence that recaptured non-captive migratory birds increase lean body mass, in addition to fat mass. This relationship persisted when we examined all recaptured transient migrants on St. George Island (Fig. S1). Interestingly, Leberg *et al.* (1996) found that after a pre-breeding trans-Gulf migratory flight, recaptured Wood Thrushes increased in body mass but did not have significantly higher lipid levels, suggesting an increase in non-fat components. Our findings corroborate the hypothesis that during stopover, initial slow increases in body mass are due to deposition of non-lipid body components, mainly protein (Carpenter *et al.,* 1993).

**Table 2:** Length of stay (mean ± SD, range in parenthesis), change in fat and lean masses between initial and final capture and rate of mass change of individual Neotropical migrants recaptured >1 day after first capture in St. George Island, FL, during pre-breeding migration 2016–2018

Species	*N*	Minimum stopover duration (days)	Change in fat (g)	Change in lean (g)	Rate of fat change (g/d)	Rate of lean change (g/d)
BLGR	2	2.00 ± 1.41 (1.00–3.00)	0.88 ± 1.73 (–0.34—2.1)	1.62 ± 1.16 (0.80–2.45)	0.18 ± 0.74 (–0.34—0.70)	0.81 ± 0.01 (0.80–0.82)
HOWA	1	1.00 ± NA (1.00–1.00)	0.04 ± NA (0.04–0.04)	−0.02 ± NA (−0.02 — −0.02)	0.04 ± NA (0.04–0.04)	−0.02 ± NA (−0.02—−0.02)
INBU	5	1.20 ± 0.45 (1.00–2.00)	0.04 ± 0.2 (–0.19—0.26)	0.06 ± 0.91 (–1.40—0.88)	0.02 ± 0.17 (–0.19—0.24)	−0.01 ± 0.85 (–1.40—0.88)
NOWA	2	1.00 ± 0.00 (1.00–1.00)	−0.04 ± 0.08 (–0.10—0.02)	2.29 ± 1.01 (1.58–3.00)	−0.04 ± 0.08 (–0.10—0.02)	2.29 ± 1.01 (1.58–3.00)
OVEN	6	2.33 ± 1.03 (1.00–4.00)	−0.06 ± 0.26 (–0.51—0.20)	0.87 ± 0.68 (–0.08—1.74)	−0.02 ± 0.13 (–0.26—0.10)	0.48 ± 0.39 (–0.02—0.95)
REVI	3	4.00 ± 3.46 (2.00–8.00)	0.62 ± 1.45 (–0.71—2.17)	0.75 ± 1.97 (–1.48—2.25)	0.04 ± 0.34 (–0.35—0.27)	0.09 ± 0.76 (–0.74—0.75)
SUTA	4	2.50 ± 1.73 (1.00–5.00)	0.18 ± 0.19 (–0.04—0.40)	0.75 ± 1.17 (–0.28—2.41)	0.07 ± 0.07 (–0.02—0.12)	0.16 ± 0.33 (–0.28—0.48)
WOTH	2	2.50 ± 2.12 (1.00–4.00)	0.96 ± 1.49 (–0.09—2.02)	2.81 ± 1.66 (1.64–3.98)	0.21 ± 0.42 (–0.09—0.51)	1.32 ± 0.45 (1.00–1.64)

The rate of mass change (g/d) for each species obtained from individuals recaptured >1 day from initial capture (fat and lean mass measured by QMR in the field).

After a trans-Gulf of Mexico pre-breeding migratory flight, birds use primarily inland forests and, if able, will overshoot coastal habitats (Buler *et al.,* 2007). Coastal habitats and barrier islands of the northern Gulf of Mexico, especially in the eastern Gulf of Mexico, are often considered as stopover habitats of “last resort” or “fire escape” stopover sites (Mehlman *et al.,* 2005). However, during fallout conditions, these sites will have high densities of migrants (Duncan, 1994) that use the habitats to rest, replenish reserves and wait for good weather (Moore and Kerlinger, 1987; Moore *et al.,* 1990; Moore and Woodrey, 1993); in this area of the Gulf of Mexico, there is a high stopover-to-passage ratio, indicating almost all birds stopover (Cohen *et al.,* 2021). Our result that migratory songbirds increase non-fat components during stopover after a trans-Gulf flight points to the importance of first-landing sites, like barrier island habitats, to the Nearctic–Neotropical migratory system.

Migration at this site in the eastern Gulf of Mexico includes long-distance migrants from Central and South America and non-breeding season birds from peninsular Florida and the Caribbean. Since we lack specific information about non-breeding origin (migratory connectivity), we used non-breeding range to examine arrival body condition. Contrary to our expectations, non-breeding range did not influence arrival fat mass or arrival lean mass. Our approach of grouping species by continental range clearly did not provide the resolution for differing non-breeding populations or differing habitat quality. Swainson’s Thrushes that spend the non-breeding season in northern Colombia exhibit different migratory departure and pace depending on habitat quality (González *et al.,* 2020), and Black-and-white Warblers (*Mniotilta varia*) that move through the Gulf of Mexico earlier spent the non-breeding season in higher quality habitat than their conspecifics that move through later (Paxton and Moore, 2015). Birds in higher quality non-breeding or staging habitat have higher departure fuel loads (Bayly *et al.,* 2016), and we would expect that the fat load a bird acquires before embarking across an ecological barrier and its body condition after crossing are inextricably linked. Migratory connectivity is a key element lacking in our understanding of the carry-over between stationary non-breeding and breeding periods. Studying the link between body condition following trans-Gulf of Mexico flight and non-breeding/staging habitat, and its subsequent effects on migratory pace and reproductive fitness, is an important future research priority to address conservation of Nearctic–Neotropical migrants. Linking migratory connectivity and physiological condition of birds during stopover may help inform the conservation and management of critical habitats and resources for trans-Gulf of Mexico migratory birds.

Fat scoring has been previously quantitatively assessed using QMR technology (Kelsey and Bairlein, 2019), and our results further confirm the utility of fat scoring in the field. However, our assessment of muscle scoring (Bairlein, 1995) using QMR showed that there was no strong relationship. A possible explanation is that muscle scoring is limited to assessing the size and shape of the pectoral muscles. However, in view of the role of lean mass deposition to the physiology of songbirds during migratory stopover, we encourage other researchers to consider muscle mass when assessing body condition of migratory songbirds. Though QMR technology may be cost prohibitive, visual pectoral muscle scoring in the field could be supplemented by measurements using a ‘muscle meter’ (Bauchinger *et al.,* 2011).

## Supplementary Material

Web_Material_coaf029

## Data Availability

The data underlying this article are available in the article and in its online supplementary material.
