# Supplementary material for: Lean mass deposition occurs at a greater rate than fat deposition during pre-breeding stopover in highly depleted songbirds in the northern Gulf of Mexico
Source: Conserv Physiol. 2025 Apr 16;13(1):coaf029. doi: 10.1093/conphys/coaf029 (PMC12002862; doi:10.1093/conphys/coaf029)

Supplemental Information for:

Lean mass deposition occurs at a greater rate than fat deposition during spring stopover in highly depleted songbirds in the northern Gulf of Mexico

Mariamar Gutierrez Ramirez[1,2*], Michael S. Griego[1,2], Joely G. DeSimone[1], Cory R. Elowe[1,2], and Alexander R. Gerson[1,2*]

1) Department of Biology, University of Massachusetts Amherst, Amherst, MA 01003, USA

2) Organismic and Evolutionary Biology Program, University of Massachusetts Amherst, Amherst, MA 01003, USA

*Co-corresponding authors:

611 N Pleasant St, 322 Morrill III, Amherst, MA 01003, USA; gutierrez.mariamar@gmail.com; argerson@umass.edu

Supplemental Data

Table S1: Migratory status and total per year for each species captured during spring migration on St. George Island, Florida.

		Year			
**Species**	**Migratory Status**	**2016**	**2017**	**2018**	**total**
Acadian Flycatcher	Nearctic-Neotropical Migrant	0	2	1	3
American Redstart	Nearctic-Neotropical Migrant	0	7	1	8
American Robin	Winter Resident	0	1	0	1
Baltimore Oriole	Nearctic-Neotropical Migrant	0	1	0	1
Bay-breasted Warbler	Nearctic-Neotropical Migrant	0	1	0	1
Black-and-white Warbler	Nearctic-Neotropical Migrant	3	3	4	10
Black-throated Blue Warbler	Nearctic-Neotropical Migrant	0	3	3	6

Blackpoll Warbler	Nearctic-Neotropical Migrant	1	1	1	3
Blue Grosbeak	Nearctic-Neotropical Migrant	1	31	9	41
Blue-headed Vireo	Nearctic-Neotropical Migrant	1	0	0	1
Blue-winged Warbler	Nearctic-Neotropical Migrant	1	0	0	1
Brown Thrasher	Year-round resident	3	3	1	7
Brown-headed Cowbird	Year-round resident	1	8	0	9
Brown-headed Nuthatch	Year-round resident	3	1	0	4
Cape May Warbler	Nearctic-Neotropical Migrant	1	0	2	3
Carolina Wren	Year-round resident	8	7	3	18
Cliff Swallow	Nearctic-Neotropical Migrant	0	1	0	1
Common Grackle	Year-round resident	7	9	0	16
Common Ground-dove	Year-round resident	1	3	0	4
Common Yellowthroat	Nearctic-Neotropical Migrant	3	7	4	14
Cooper's Hawk	Winter Resident	0	1	0	1
Downy Woodpecker	Year-round resident	0	2	2	4
Eastern Towhee	Year-round resident	6	0	2	8
Eurasian Collard-Dove	Year-round resident	0	1	0	1
Gray Catbird	Nearctic-Neotropical Migrant	95	122	96	313
Gray Kingbird	Year-round resident	1	0	0	1
Gray-cheeked Thrush	Nearctic-Neotropical Migrant	0	2	15	17
Great-crested Flycatcher	Nearctic-Neotropical Migrant	3	2	4	9
Hermit Thrush	Winter Resident	1	0	0	1
Hooded Warbler	Nearctic-Neotropical Migrant	3	4	29	36
House Finch	Year-round resident	0	1	0	1
Indigo Bunting	Nearctic-Neotropical Migrant	8	28	24	60
Kentucky Warbler	Nearctic-Neotropical Migrant	0	1	6	7
Louisiana Waterthrush	Nearctic-Neotropical Migrant	0	1	0	1
Mourning Dove	Year-round resident	1	1	0	2
Northern Cardinal	Year-round resident	29	16	2	47
Northern Mockingbird	Year-round resident	3	7	0	10
Northern Parula	Nearctic-Neotropical Migrant	0	1	0	1
Northern Waterthrush	Nearctic-Neotropical Migrant	32	17	5	54
Orchard Oriole	Nearctic-Neotropical Migrant	0	0	1	1

Ovenbird	Nearctic-Neotropical Migrant	22	19	18	59
Painted Bunting	Nearctic-Neotropical Migrant	0	3	8	11
Prairie Warbler	Nearctic-Neotropical Migrant	2	1	0	3
Prothonotary Warbler	Nearctic-Neotropical Migrant	12	2	4	18
Red-bellied Woodpecker	Year-round resident	5	1	0	6
Red-eyed Vireo	Nearctic-Neotropical Migrant	9	12	14	35
Red-winged Blackbird	Year-round resident	1	9	0	10
Rose-breasted Grosbeak	Nearctic-Neotropical Migrant	0	7	1	8
Ruby-crowned kinglet	Winter Resident	0	1	1	2
Ruby-throated Hummingbird	Nearctic-Neotropical Migrant	4	7	0	11
Scarlet Tanager	Nearctic-Neotropical Migrant	4	3	4	11
Summer Tanager	Nearctic-Neotropical Migrant	3	8	16	27
Swainson's Thrush	Nearctic-Neotropical Migrant	4	15	9	28
Swainson's Warbler	Nearctic-Neotropical Migrant	3	5	1	9
Swamp Sparrow	Winter Resident	0	1	0	1
Tennessee Warbler	Nearctic-Neotropical Migrant	0	0	1	1
Veery	Nearctic-Neotropical Migrant	3	4	19	26
Western Palm Warbler	Winter Resident	8	2	0	10
White-eyed Vireo	Nearctic-Neotropical Migrant	5	2	18	25
Wood Thrush	Nearctic-Neotropical Migrant	9	8	22	39
Worm-eating Warbler	Nearctic-Neotropical Migrant	4	6	6	16
Yellow Warbler	Nearctic-Neotropical Migrant	0	2	0	2
Yellow-bellied Sapsucker	Winter Resident	0	0	1	1
Yellow-billed Cuckoo	Nearctic-Neotropical Migrant	7	4	3	14
Yellow-breasted Chat	Nearctic-Neotropical Migrant	1	0	1	2
Yellow-rumped Warbler	Winter Resident	0	1	4	5
Yellow-throated Vireo	Nearctic-Neotropical Migrant	1	2	1	4
Yellow-throated Warbler	Nearctic-Neotropical Migrant	1	0	1	2
		324	421	368	1113
					1113

Table S2: Average (± SD) and range of fat mass, lean mass, and body mass of songbirds captured in spring on St. George Island, Florida. Fat mass and lean mass measurements taken by quantitative magnetic resonance technology and are uncorrected for body size. Body mass taken by digital scale to the 0.01 g.

Species	*N*	Fat (g)	Lean (g)	Body mass (g)
Acadian Flycatcher	2	0.42 ± 0.24<br>0.26-0.59	8.91 ± 1.98<br>7.51-10.31	11.72 ±2.19<br>10.17-13.27
American Redstart	7	0.49 ±0.36<br>0.00-0.95	5.83 ±0.35<br>5.25-6.39	7.53 ±0.63<br>6.68-8.21
American Robin	1	7.42 ±NA<br>7.42-7.42	60.38 ±NA<br>60.38-60.38	78.62 ±NA<br>78.62-78.62
Baltimore Oriole	1	2.56 ±NA<br>2.56-2.56	24.14 ±NA<br>24.14-24.14	31.50 ±NA<br>31.50-31.50
Bay-breasted Warbler	1	0.34 ±NA<br>0.34-0.34	7.56 ±NA<br>7.56-7.56	9.75 ±NA<br>9.75-9.75
Black-and-white Warbler	9	0.47 ±0.45<br>0.01-1.44	6.91 ±0.98<br>4.72-8.01	9.22 ±0.94<br>7.74-10.63
Black-throated Blue Warbler	5	0.35 ±0.45<br>0.00-1.07	6.18 ±0.53<br>5.62-6.71	7.91 ±0.62<br>7.15-8.74
Blackpoll Warbler	3	0.17 ±0.19<br>0.00-0.38	5.75 ±3.57<br>1.71-8.45	11.04 ±1.70<br>10.03-13.00
Blue Grosbeak	40	2.27 ±2.83<br>0.00-10.33	19.51 ±2.34<br>10.31-23.47	26.13 ±4.10<br>20.42-34.83
Blue-headed Vireo	1	0.56 ±NA<br>0.56-0.56	13.74 ±NA<br>13.74-13.74	16.74 ±NA<br>16.74-16.74
Blue-winged Warbler	1	0.55 ±NA<br>0.55-0.55	5.23 ±NA<br>5.23-5.23	7.17 ±NA<br>7.17-7.17
Brown Thrasher	3	2.54 ±1.28<br>1.06-3.35	55.45 ±4.70<br>50.66-60.05	67.41 ±5.76<br>61.17-72.52

Species	N	Fat (g)	Lean (g)	Body mass (g)
Brown-headed Cowbird	4	2.30 ±0.65<br>1.49-3.02	30.68 ±2.95<br>27.70-34.76	38.44 ±2.93<br>34.90-41.90
Brown-headed Nuthatch	1	2.51 ±NA<br>2.51-2.51	6.47 ±NA<br>6.47-6.47	9.83 ±NA<br>9.83-9.83
Cape May Warbler	3	1.05 ±1.01<br>0.14-2.14	6.82 ±0.43<br>6.34-7.17	9.55 ±1.10<br>8.45-10.65
Carolina Wren	9	0.19 ±0.21<br>0.00-0.57	16.12 ±1.60<br>13.14-17.93	19.45 ±2.47<br>16.17-24.72
Common Grackle	7	1.63 ±1.36<br>0.28-3.96	79.56 ±15.17<br>63.70-101.44	95.30 ±17.33<br>77.50-119.59
Common Yellowthroat	11	0.47 ±0.36<br>0.04-1.18	7.11 ±0.50<br>6.43-8.02	9.25 ±0.90<br>8.12-11.13
Eastern Towhee	7	1.31 ±1.80<br>0.00-4.82	33.04 ±4.45<br>24.03-36.96	41.63 ±2.87<br>37.39-44.96
Gray Catbird	288	3.00 ±2.79<br>0.00-15.82	26.21 ±2.98<br>21.3-36.19	34.46 ±3.72<br>26.93-46.85
Gray Kingbird	1	1.59 ±NA<br>1.59-1.59	37.44 ±NA<br>37.44-37.44	45.63 ±NA<br>45.63-45.63
Gray-cheeked Thrush	17	1.35 ±1.00<br>0.04-2.90	18.81 ±1.74<br>15.95-22.50	24.50 ±2.39<br>20.03-29.64
Great-crested Flycatcher	7	0.69 ±0.70<br>0.03-1.95	30.56 ±2.19<br>27.33-33.37	31.80 ±12.06<br>5.09-39.04
Hooded Warbler	28	0.52 ±0.51<br>0.00-2.02	7.14 ±0.94<br>4.11-8.52	9.48 ±0.86<br>7.96-11.51
House Finch	1	0.76 ±NA<br>0.76-0.76	15.66 ±NA<br>15.66-15.66	18.78 ±NA<br>18.78-18.78
Indigo Bunting	53	0.81 ±0.80<br>0.02-3.42	9.31 ±1.22<br>6.71-11.99	12.82 ±1.33<br>10.84-15.42
Kentucky Warbler	4	0.17 ±0.14<br>0.00-0.29	9.26 ±0.77<br>8.16-9.82	11.30 ±0.61<br>10.60-12.05

Species	*N*	Fat (g)	Lean (g)	Body mass (g)
Louisiana Waterthrush	1	0.10 ±NA<br>0.10-0.10	9.16 ±NA<br>9.16-9.16	15.81 ±NA<br>15.81-15.81
Mourning Dove	1	2.88 ±NA<br>2.88-2.88	102.97 ±NA<br>102.97-102.97	122.74 ±NA<br>122.74-122.74
Northern Cardinal	37	0.91 ±0.54<br>0.01-2.12	30.22 ±5.54<br>0.06-35.56	38.44 ±2.20<br>33.66-44.44
Northern Mockingbird	7	1.99 ±1.61<br>0.00-4.41	39.48 ±3.11<br>36.68-45.67	48.85 ±3.71<br>44.56-55.55
Northern Parula	1	0.44 ±NA<br>0.44-0.44	5.82 ±NA<br>5.82-5.82	7.39 ±NA<br>7.39-7.39
Northern Waterthrush	52	1.52 ±1.42<br>0.13-5.38	10.53 ±1.78<br>6.45-13.24	15.08 ±1.87<br>12.42-19.79
Orchard Oriole	1	4.08 ±NA<br>4.08-4.08	15.64 ±NA<br>15.64-15.64	22.42 ±NA<br>22.42-22.42
Ovenbird	49	0.97 ±1.13<br>0.00-5.38	12.25 ±1.74<br>6.29-15.47	16.54 ±1.63<br>13.48-21.21
Painted Bunting	11	0.87 ±0.62<br>0.00-1.86	11.53 ±0.90<br>10.66-13.02	14.94 ±1.12<br>13.56-16.59
Prairie Warbler	2	0.84 ±0.51<br>0.48-1.20	4.62 ±0.30<br>4.41-4.83	7.08 ±0.37<br>6.82-7.34
Prothonotary Warbler	17	1.13 ±0.83<br>0.23-3.56	8.03 ±2.00<br>4.43-11.17	13.19 ±1.70<br>11.18-17.31
Red-bellied Woodpecker	1	1.87 ±NA<br>1.87-1.87	56.87 ±NA<br>56.87-56.87	63.51 ±NA<br>63.51-63.51
Red-eyed Vireo	32	1.30 ±1.43<br>0.00-5.87	10.83 ±1.58<br>7.43-13.83	15.11 ±1.98<br>11.87-21.64
Red-winged Blackbird	1	0.92 ±NA<br>0.92-0.92	52.37 ±NA<br>52.37-52.37	61.47 ±NA<br>61.47-61.47
Rose-breasted Grosbeak	8	2.25 ±1.53<br>0.08-4.39	30.48 ±2.31<br>26.48-33.09	39.02 ±3.65<br>32.58-43.88

Species	*N*	Fat (g)	Lean (g)	Body mass (g)
Ruby-crowned kinglet	1	0.18 ±NA<br>0.18-0.18	4.61 ±NA<br>4.61-4.61	5.71 ±NA<br>5.71-5.71
Ruby-throated Hummingbird	1	0.48 ±NA<br>0.48-0.48	2.48 ±NA<br>2.48-2.48	3.29 ±NA<br>3.29-3.29
Scarlet Tanager	10	1.89 ±2.20<br>0.00-5.95	19.40 ±1.52<br>16.40-21.37	25.68 ±2.95<br>20.75-30.81
Summer Tanager	26	1.42 ±1.22<br>0.01-4.96	20.72 ±1.43<br>18.45-23.24	26.62 ±2.26<br>23.48-32.59
Swainson's Thrush	28	1.24 ±0.97<br>0.00-3.42	20.31 ±1.42<br>17.30-22.67	25.69 ±1.83<br>21.63-28.64
Swainson's Warbler	9	0.75 ±0.53<br>0.17-1.51	9.32 ±1.99<br>6.45-12.41	13.13 ±1.09<br>11.37-14.65
Tennessee Warbler	1	1.41 ±NA<br>1.41-1.41	6.74 ±NA<br>6.74-6.74	8.88 ±NA<br>8.88-8.88
Veery	22	1.70 ±1.27<br>0.03-4.71	19.66 ±1.34<br>16.98-22.16	25.39 ±2.31<br>20.97-29.66
Western Palm Warbler	8	0.36 ±0.26<br>0.00-0.81	6.43 ±1.27<br>4.48-8.07	9.01 ±0.66<br>8.32-10.24
White-eyed Vireo	22	1.09 ±0.74<br>0.04-2.52	7.75 ±1.15<br>5.09-10.09	10.93 ±1.02<br>8.95-12.90
Wood Thrush	37	2.60 ±3.22<br>0.16-13.81	30.83 ±2.60<br>23.75-37.92	40.40 ±4.20<br>33.81-52.39
Worm-eating Warbler	13	0.47 ±0.31<br>0.00-1.00	8.93 ±0.77<br>7.76-10.06	11.30 ±1.00<br>8.78-12.32
Yellow Warbler	2	0.41 ±0.46<br>0.09-0.74	6.53 ±0.10<br>6.46-6.60	8.64 ±0.71<br>8.14-9.14
Yellow-billed Cuckoo	14	1.02 ±1.37<br>0.00-4.07	32.20 ±1.91<br>28.32-34.77	42.23 ±2.78<br>36.65-45.78
Yellow-breasted Chat	1	0.97 ±NA<br>0.97-0.97	16.79 ±NA<br>16.79-16.79	21.23 ±NA<br>21.23-21.23

Species	*N*	Fat (g)	Lean (g)	Body mass (g)
Yellow-rumped Warbler	3	0.92 ±0.17<br>0.77-1.11	8.98 ±0.78<br>8.12-9.64	12.11 ±0.15<br>11.97-12.27
Yellow-throated Vireo	4	2.06 ±2.04<br>0.45-5.04	10.64 ±2.79<br>7.86-13.66	16.93 ±2.83<br>14.89-21.04
Yellow-throated Warbler	2	1.40 ±0.19<br>1.26-1.53	7.56 ±0.33<br>7.33-7.79	10.59 ±0.43<br>10.29-10.90

Figure S1: Rate of fat mass and lean mass change relative to minimum stopover duration of 15 transient Neotropical migrant species ($N = 34$) recaptured >1 day after first capture in St. George Island, Florida during spring migration 2016-2018. Fat and lean mass measured by Quantitative magnetic resonance in the field. The dashed horizontal line indicates no change in mass between first capture and subsequent recapture, so that below the line in red are birds with negative rate and above the line in black are birds with positive rate of mass change.

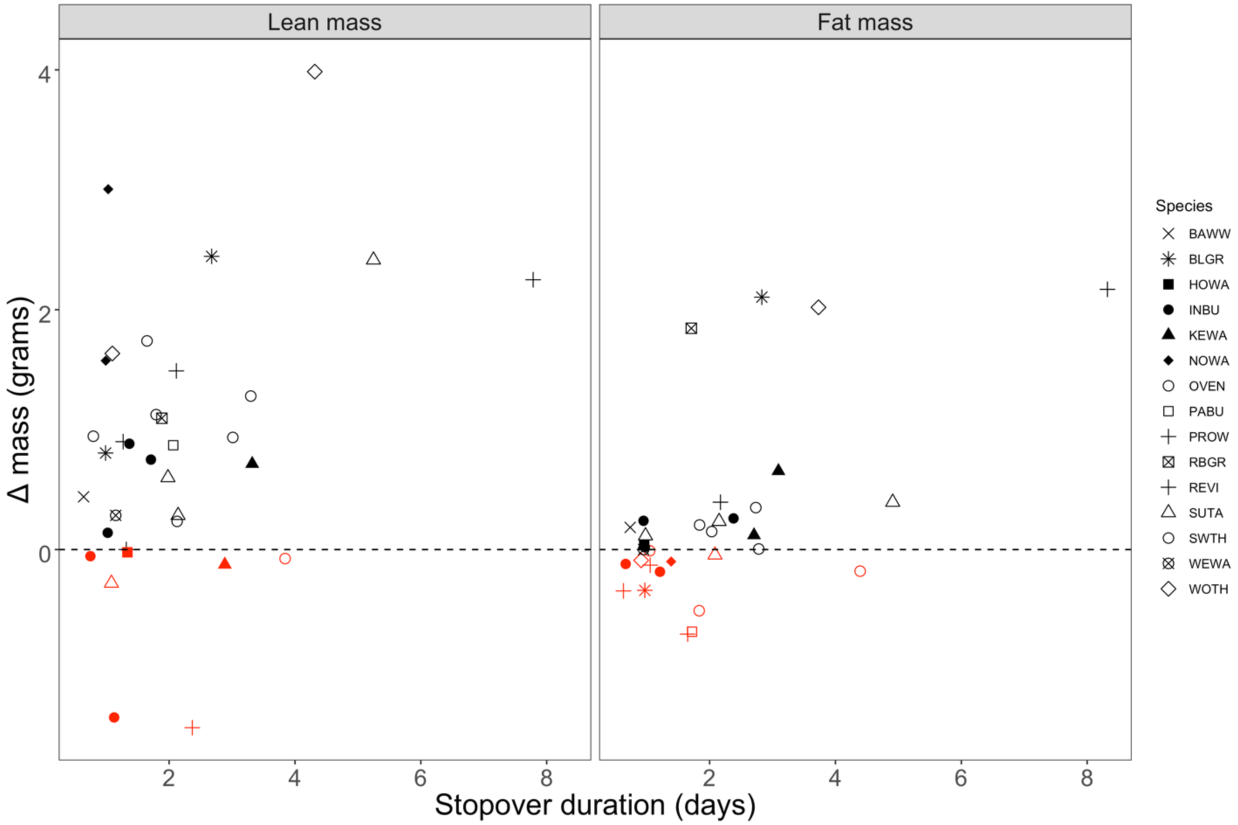

Supplement: Web_Material_coaf029 [file web_material_coaf029.pdf]
